# A survey of tobacco dependence treatment guidelines content in 61 countries

**DOI:** 10.1111/add.14204

**Published:** 2018-04-16

**Authors:** Kapka Nilan, Ann McNeill, Rachael L. Murray, Tricia M. McKeever, Martin Raw

**Affiliations:** ^1^ UK Centre for Tobacco and Alcohol Studies, School of Medicine University of Nottingham Nottingham UK; ^2^ UK Centre for Tobacco and Alcohol Studies Institute of Psychiatry, Psychology and Neuroscience (IoPPN), King's College London London UK; ^3^ New York University College of Global Public Health and NYU Medical School New York NY USA

**Keywords:** FCTC Article 14, high, middle‐ and low‐income countries, tobacco dependence treatment guidelines, tobacco control, WHO FCTC

## Abstract

**Aims:**

To assess tobacco dependence treatment guidelines content in accordance with Article 14 of the World Health Organization (WHO) Framework Convention on Tobacco Control (FCTC) and its guidelines, and association between content and country income level.

**Design:**

Cross‐sectional study.

**Setting:**

On‐line survey from March to July 2016.

**Participants:**

Contacts in 77 countries, including 68 FCTC Parties, six Signatories and three non‐Parties which had indicated having guidelines in previous surveys, or had not been surveyed before.

**Measurements:**

A nine‐item questionnaire on guidelines content, key recommendations, writing and dissemination.

**Findings:**

We received responses from contacts in 63 countries (82%); 61 had guidelines. The majority are for doctors (93%), primary care (92%) and nurses (75%). All recommend brief advice, 82% recording tobacco use in medical notes, 98% nicotine replacement therapy (NRT), 61% quitlines, 31% text messaging and 87% intensive specialist support, and 54% stress the importance of health‐care workers not using tobacco. Only 57% have a dissemination strategy, and 62% have not been updated for 5 or more years. Compared with high‐income countries, quitlines are less likely to be recommended in upper middle‐income countries guidelines [odds ratio (OR) = 0.15, 95% confidence interval (CI) = 0.04–0.61] and intensive specialist support in lower middle‐income countries guidelines (OR = 0.01, 95% CI = 0.00–0.20). Guidelines updating is associated positively with country income level (*P* = 0.027).

**Conclusions:**

Although most tobacco dependence treatment guidelines in the 61 countries assessed in 2016 follow the World Health Organization's Framework Convention on Tobacco Control Article 14 recommendations and do not differ significantly by income level, improvements are needed in keeping guidelines up‐to‐date, applying good writing practices and developing a dissemination strategy.

## Introduction

Article 14 of the World Health Organization (WHO) Framework Convention on Tobacco Control (FCTC) requires Parties to develop and disseminate comprehensive guidelines based on scientific evidence, and to take effective measures to promote cessation of tobacco use and adequate treatment for tobacco dependence 1.

Guidelines for the implementation of Article 14, adopted at the fourth Conference of the Parties to the FCTC in 2010, provide more detailed recommendations and outline the key characteristics of national tobacco treatment guidelines 2 which should:

- be evidence‐based;
- be comprehensive, and include a broad range of interventions; for example, brief advice, quitlines, behavioural support and cessation medications;
- cover all settings and providers, both within and outside the health‐care systems;
- include a dissemination and implementation plan;
- stress the importance of all service providers setting an example by not using tobacco, and that they be offered help to stop;
- be developed in active collaboration with key stakeholders, including health professional organizations;
- be endorsed widely at national level by health professional organizations and/or associations;
- be protected from all actual and potential conflicts of interest; and
- be reviewed periodically and updated in light of new scientific evidence.

Previous surveys identified 31 guidelines in 2007 and 53 in 2012 3, 4. In our most recent (2015) global survey of tobacco treatment provision, in 142 countries, we identified 57 guidelines 5. The treatment survey also found that more than half of high‐income countries had guidelines (63%), while fewer than half of middle‐income and only one low‐income country (LIC) did 5, showing a strong association between having guidelines and country income‐level. It is not known if this income gradient is reflected in the content of the guidelines and the way countries with different income levels follow the recommendations of Article 14 and its guidelines.

In this paper we report the results of a survey of the content of the guidelines, with the aims of (1) assessing how well they follow the recommendations of the 2010 FCTC Article 14 guidelines; and (2) assessing the association between guideline content and country income‐level.

## Methods

### Survey participants

We approached all Parties (*n* = 68) and Signatories (*n* = 2) that confirmed having treatment guidelines in any of the previous surveys. We also tried to reach another 14 countries that were not Parties or Signatories at the time of the survey and were not surveyed previously, and found contacts in seven of them. We did not approach Parties that said they did not have guidelines in 2015 (*n* = 85) and non‐respondents (*n* = 30) in the 2015 survey. The reason for excluding the non‐respondents was that they did not reply to our multiple attempts to contact them previously.

Our final survey sample consisted of 64 FCTC Parties plus the United Kingdom (one Party but comprising four countries; England, Scotland, Northern Ireland and Wales, each with their own health‐care system and therefore surveyed individually), six Signatories and three non‐Parties at the time of the survey, a total of 77 countries (Supporting information, Table S1). We did not find contacts for one Signatory and six non‐Parties.

For the countries surveyed before we invited the same contacts from our previous treatment survey 5 to take part by e‐mail. For countries not surveyed before, we identified contacts with the help of the WHO regional offices, the Framework Convention Alliance (FCA), Action on Smoking and Health (USA) and other professional networks and contacts. The contacts were a mixture of tobacco control government officials, members of non‐governmental health organizations and tobacco cessation treatment specialists.

The questionnaire, adapted from our previous survey, contained nine items based on the Article 14 guidelines key recommendations. We sent a draft to around 10 international tobacco treatment experts known to the authors, most of whom helped to design the previous survey questionnaire. They included researchers and government officials. We amended the questionnaire in line with their feedback. The final version included questions on the guidelines evidence base, evidence‐based interventions, who the guidelines were for, cessation medications, the writing and review process, funding and dissemination.

We e‐mailed our contacts in March 2016 and asked them to complete the questionnaire in an attached Word file (available in English, French, Spanish and Russian) or on‐line (English only). We sent reminder e‐mails to non‐respondents every 2 weeks between March and June 2016. Where responses were incomplete or unclear, we asked for additional information or clarification. The survey was closed at the end of July 2016.

Survey responses were analysed by countries’ World Bank (WB) income classification on 1 July 2015 6. We used the Cochrane Armitage statistic to test association between guideline content and income level and explored significant findings using logistic regression.

The statistical analyses were conducted in Stata version 14. In order to assess how recently guidelines had been updated, we checked the year of publication of previous versions for each country, using data from our 2007, 2012 and 2015 surveys 3, 4, 5.

We use the following abbreviations for income level: high‐income countries (HIC), upper middle‐income countries (UMIC), lower middle‐income countries (LMIC) and low‐income countries (LIC).

## Results

### Survey response rate

We received responses from contacts in 63 of the 77 countries (82%). Two reported not having guidelines, and the remaining 61 included 57 Parties and four Signatories that reported having guidelines and completed the questionnaire (see Supporting information, Table S1). A list of countries with guidelines is also available on‐line (see Supporting information, Table S2).

### Guidelines by region and income level

Sixty‐four per cent of the 61 countries with guidelines were HIC, 21% UMIC and 15% LMIC. No LIC reported having guidelines. By WHO region, 56% were in Europe, 18% in the Americas, 13% in the Western Pacific, 10% in the Eastern Mediterranean, 3% in South East Asia and none in Africa.

### Guideline content

#### Key recommendations

All guidelines recommend brief advice, 82% recommend recording tobacco use in medical notes, 98% recommend smoking cessation medications, 61% recommend quitlines, 31% recommend text messaging, 87% recommend intensive specialist support and 54% stress the importance of health‐care workers (HCWs) setting an example by not using tobacco (Table 1). Quitlines were less likely to be recommended in UMIC compared with HIC [odds ratio (OR) = 0.15, 95% confidence interval (CI) = 0.04–0.61], while intensive specialist support was less likely to be recommended in LMIC compared with HIC (OR = 0.01, 95% CI = 0.00–0.20) (Supporting information, Table S3).

**Table 1:** Guidelines content by World Bank income level, % Yes (n).

Question	All (61)	HIC (39)	UMIC (13)	LMIC (9)	P‐value for trend
Do the guidelines recommend brief advice?	100 (61)	100 (39)	100 (13)	100 (9)	–
Do the guidelines recommend recording tobacco use in patients’ medical notes?	82 (50)	85 (33)	69 (9)	89 (8)	0.855
Do the guidelines recommend quitlines?	61 (37)	74 (29)	31 (4)	44 (4)	0.017^a^
Do the guidelines recommend text messaging?	31 (19)	33 (13)	31 (4)	22 (2)	0.539
Do the guidelines recommend intensive specialist support?	87 (53)	97 (38)	92 (12)	33 (3)	< 0.001^b^
Do the guidelines stress the importance of health‐care workers setting an example by not using tobacco?	54 (33)	51 (20)	69 (9)	31 (4)	0.937
Do the guidelines include evidence on cost‐effectiveness?	54 (33)	59 (23)	62 (8)	22 (2)	0.100
Do the guidelines reference or refer to the Cochrane Library?	66 (40)	74 (29)	77 (10)	11 (1)	0.003^c^
Do the guidelines reference or refer to the guidelines of other countries?	72 (44)	67 (26)	92 (12)	67 (6)	0.529
Are the guidelines based on another country's guidelines or other guidelines?	59 (36)	49 (19)	77 (10)	78 (7)	0.094
Do the guidelines recommend NRT?	98 (60)	100 (39)	92 (12)	100 (9)	0.505
Do the guidelines recommend bupropion?	84 (51)	92 (36)	77 (10)	56 (5)	0.008^d^
Do the guidelines recommend varenicline?	82 (50)	87 (34)	75 (9)	78 (7)	0.354
Do the guidelines recommend cytisine?	11 (7)	5 (2)	8 (1)	44 (4)	0.003^e^

HIC = high‐income countries; UMIC = upper middle‐income countries; LMIC = lower middle‐income countries; NRT = nicotine replacement therapy.

Odds ratios (OR), 95% confidence interval (CI) where *P*‐value for trend is significant.

a HIC = 1: UMIC, OR = 0.15, 95% CI = 0.04–0.61; LMIC: OR = 0.28, 95% CI = 0.06–1.23;

b HIC = 1; UMIC, OR = 0.32, 95% CI = 0.01–5.44; LMIC: OR = 0.01, 95% CI = 0.00–0.20;

c HIC =1: UMIC, OR = 1.15, 95% CI = 0.26–5.03; LMIC: OR = 0.04, 95% CI = 0.00–0.39;

d HIC = 1: UMIC, OR = 0.42, 95% CI = 0.06–2.84; LMIC: OR = 0.10, 95% CI = 0.02–0.61;

e HIC = 1: UMIC, OR = 0.16, 95% CI = 0.30–0.80; LMIC: OR = 14.8, 95% CI = 2.13–102.71.

#### Smoking cessation medications

Almost all guidelines, 98%, recommend nicotine replacement therapy (NRT), 84% recommend bupropion, 82% recommend varenicline and 11% recommend cytisine (Table 1). Bupropion was less likely to be recommended in LMIC compared with HIC (OR = 0.10, 95% CI = 0.02–0.61), while cytisine was more likely to be recommended (OR = 14.8, 95% CI = 2.13–102.71) (Supporting information, Table S3).

#### Other content

Around two‐thirds (66%) reference or refer to the Cochrane Library, 72% reference or refer to guidelines of other countries and 59% are based on another country's guidelines or other guidelines; just over half (54%) of guidelines include evidence of cost‐effectiveness (Table 1). Compared to HIC, LMIC guidelines were less likely to contain references to the Cochrane Library (OR = 0.04, 95% CI = 0.00–0.39) (Supporting information, Table S3).

#### Professions, settings and client groups the guidelines were for

Almost all guidelines, 92%, are for primary care, and just over three‐quarters for health service managers and smoking cessation specialists; 93% are for doctors, 75% for nurses, 53% for dentists and 48% for pharmacists. Two‐thirds (67%) include hospitals, 53% addiction services and 48% mental health services; 76% cover pregnant tobacco users and 23% smokeless tobacco users. UMIC and LMIC country guidelines are likely to cover fewer health‐care professionals and settings compared with HIC. The trend is significant for doctors (*P* = 0.039), nurses (*P* = 0.001), pharmacists (*P* < 0.001), dentists (*P* = 0.012), smoking cessation specialists (*P* = 0.046) and primary care (*P* = 0.030). A similar trend was observed with mental health services (*P* = 0.048) and pregnant tobacco users (*P* = 0.044) in UMIC and LMIC (Table 2). Compared with HIC, LMIC guidelines were less likely to cover nurses (OR = 0.07, 95% CI = 0.01–0.40) and dentists (OR = 0.16, 95% CI = 0.03–0.87) (Supporting information, Table S4).

**Table 2:** Professions, settings and client groups covered in the guidelines, % yes (n).

Question	All (61)	HIC (39)	UMIC (13)	LMIC (9)	P‐value for trend
Which professions, settings, and client groups do they include:					
Doctors?	93 (57)	97 (38)	92 (12)	78 (7)	0.039
Nurses?	75 (46)	87 (34)	69 (9)	33 (3)	0.001^a^
Pharmacists?	48 (29)	62 (24)	31 (4)	11 (1)	< 0.001^b^
Dentists?	53 (32)	64 (25)	39 (5)	22 (2)	0.012^c^
Smoking cessation specialists?	77 (47)	85 (33)	69 (9)	56 (5)	0.046^d^
Health‐care service managers?	76 (35)	59 (23)	62 (8)	31 (4)	0.534
Primary care?	92 (56)	97 (38)	85 (11)	78 (7)	0.030^e^
Hospitals?	67 (41)	72 (28)	69 (9)	31 (4)	0.160
Mental health services?	48 (29)	56 (22)	39 (5)	22 (2)	0.048^f^
Addiction services?	53 (32)	59 (23)	39 (5)	31 (4)	0.261
Prisons?	15 (9)	21 (8)	8 (1)	0 (0)	0.083
Smokeless tobacco users?	23 (14)	28 (11)	15 (2)	11 (1)	0.203
Pregnant tobacco users?	76 (35)	67 (26)	46 (6)	33 (3)	0.044^g^
Other?	25 (15)	21 (8)	39 (5)	22 (2)	0.582

HIC = high‐income countries; UMIC = upper middle‐income countries; LMIC = lower middle‐income countries.

Odds ratios (OR), 95% confidence interval (CI) where *P*‐value for trend is significant.

a HIC = 1: UMIC, OR = 0.33, 95% CI = 0.07–1.49; LMIC, OR = 0.07, 95% CI = 0.01–0.40;

b HIC = 1: UMIC, OR = 0.27, 95% CI = 0.07–1.06; logistic regression not possible for LMIC, as only one value;

c HIC = 1: UMIC, OR = 0.35, 95% CI = 0.10–1.28; LMIC, OR = 0.16, 95% CI = 0.03–0.87;

d HIC =1, UMIC OR 0.41 (95% CI 0.09 – 1.77); LMIC OR 0.23 (95% CI 0.05 – 1.10);

e HIC = 1: UMIC, OR = 0.14, 95% CI = 0.01–1.76; LMIC, OR = 0.09, 95% CI = 0.01–1.16;

f HIC =1: UMIC, OR = 0.48, 95% CI = 0.13–1.74; LMIC, OR = 0.22, 95% CI = 0.04–1.20;

g HIC =1: UMIC, OR = 0.43, 95% CI = 0.12–1.54; LMIC, OR = 0.25, 95% CI = 0.05–1.16.

### Guidelines writing, dissemination and funding

Sixty‐one per cent of guidelines describe the writing and review process clearly. National professional associations participated in the writing and review process of 77% of guidelines, and 66% are endorsed formally by them; 67% are formally endorsed or supported by the national government, and 61% were peer‐reviewed. Sixty‐nine per cent are published on‐line, 74% as a book or report and 21% in a peer‐reviewed journal. Forty‐one per cent of guidelines include conflict‐of‐interest statements for all authors; 69% received financial support from government or other public health organization, 13% received financial support from the pharmaceutical industry and 8% include the names and/or logos of pharmaceutical companies (Table 3). Fifty‐seven per cent had a dissemination strategy. Compared with HIC, UMIC guidelines were less likely to contain conflict‐of‐interest statements (OR = 0.16, 95% CI = 0.30–0.80) (Supporting information, Table S3).

**Table 3:** Guidelines writing and review process by World Bank income level, % Yes (n).

Question	All (61)	HIC (39)	UMIC (13)	LMIC (9)	P‐value for trend
Do the guidelines clearly describe the writing and review process?	61 (37)	66 (25)	62 (8)	44 (4)	0.268
Did national professional associations participate in drafting or reviewing them?	77 (47)	80 (31)	77 (10)	67 (6)	0.441
Are they formally endorsed by national professional associations?	66 (40)	67 (26)	62 (8)	67 (6)	0.906
Are they formally endorsed or supported by your national government?	67 (41)	67 (26)	62 (8)	78 (7)	0.670
Were they peer‐reviewed?	61 (37)	67 (26)	62 (8)	38 (3)	0.122
Do they include conflict‐of‐interest statements for all authors?	41 (25)	54 (21)	15 (2)	22 (2)	0.019^a^
Is there a strategy to disseminate the guidelines?	57 (35)	66 (25)	54 (7)	33 (3)	0.075
Where are the guidelines published?					
In a peer‐reviewed scientific journal	21 (13)	26 (10)	23 (3)	0 (0)	
As a report/book	74 (45)	74 (29)	85 (11)	56 (5)	
On‐line	69 (42)	74 (29)	69 (9)	50 (4)	
Other	3 (2)	0 (0)	0 (0)	22 (2)	
Do they state clearly who funded the guidelines?	66 (40)	72 (28)	54 (7)	56 (5)	0.179
Did they receive financial support from government or other public health organizations?	69 (42)	72 (28)	62 (8)	67 (6)	0.429
Did they receive financial support from the pharmaceutical industry?	13 (8)	14 (6)	0 (0)	22 (2)	0.863
Do the names and/or logos of any pharmaceutical companies appear in the guidelines?	8 (5)	11 (4)	0 (0)	11 (1)	0.716

HIC = high‐income countries; UMIC = upper middle‐income countries; LMIC = lower middle‐income countries. Odds ratios (OR), 95% confidence interval (CI) where *P*‐value for trend is significant.

a HIC = 1; UMIC, OR= 0.16, 95% CI= 0.30 – 0.80.

### Year of publishing and updating guidelines

We were uncertain of the year of publication of one country's guidelines and excluded it from the analyses, leaving 60 countries that confirmed year of publishing and/or updating. Of these 60, five (8%) published guidelines for the first time since 2012, 18 (30%) had updated their guidelines since 2012 and 37 (62%) had not updated their guidelines since 2012, including all LMIC, 75% of UMIC and 58% of HIC. Guidelines updating was associated positively with income level (*P*‐value for trend = 0.027).

Compared with guidelines not updated since 2012, more recently updated or written guidelines recommended quitlines (83% compared with 49%, *P* = 0.007) and intensive cessation support (100% compared with 78%, *P* = 0.024) (Table 4).

**Table 4:** Comparison between guidelines published or updated before and after 2012.

Guideline recommendations, % Yes (n)	Guidelines updated before 2012 (n = 37)	Guidelines updated or published after 2012 (n = 23)	P‐value for difference
Recording tobacco use in medical notes	76 (28)	91 (21)	*P* = 0.181
Brief advice	100 (37)	100 (23)	–
NRT	100 (37)	100 (23)	–
Bupropion	81 (30)	91 (21)	*P* = 0.133
Varenicline	76 (28)	91 (21)	*P* = 0.059
Cytisine	14 (5)	10 (2)	*P* = 0.654
Specialized support	78 (29)	100 (23)	*P* = 0.024
Quitlines	49 (18)	83 (19)	*P* = 0.007
Text messaging	24 (9)	44 (10)	*P* = 0.192
Stress the importance of health‐care workers setting an example by not using tobacco	53 (20)	57 (13)	*P* = 0.982

NRT = nicotine replacement therapy.

## Discussion

We identified 61 countries with treatment guidelines, five of which had produced them since 2012. The guidelines are distributed disproportionately by income level and region, as in our 2012 survey 4. More than two‐thirds are high‐income, one‐third middle‐income; and no low‐income countries had guidelines. More than half are in Europe and fewer than 20% in each of the other WHO regions, except Africa, which still has no country with guidelines. As in the 2012 survey 4 most guidelines are broadly evidence‐based. All recommend brief advice and almost all recommend smoking cessation medications. More than 80% recommend recording tobacco use in medical notes and intensive specialist support, and more than half stress the importance of health‐care workers not using tobacco. Fewer than two‐thirds recommend quitlines and fewer than one‐third recommend text messaging. There were important differences in guidelines content by income; for example, middle‐income country guidelines covered fewer health‐care professionals and settings compared with HIC.

### Strengths and limitations

To our knowledge, this is the most comprehensive survey of national tobacco treatment guidelines to date. We identified contacts in all countries that previously said they had guidelines, as well as in additional countries for which such information was not available at the time of the survey.

For this survey we tried to identify all countries with guidelines. We did not approach countries that did not respond or responded that they did not have guidelines in the 2015 survey. As the majority were low‐ and lower‐middle income, it was highly unlikely for these countries to have developed treatment guidelines during the short period of time (just 1 year) between the two surveys. Of the 30 countries not included in the survey, 17 were low‐ or lower middle‐income, 10 upper middle‐income and three high‐income. Although it can be speculated that it is possible for some of these countries to have had, or to have consequently developed, treatment guidelines, of the 12 that responded in the 2012 survey only two reported guidelines. The remaining 18 countries did not respond to any of our previous surveys (2012 and 2015); hence, we have been unable to verify their guidelines status.

Except for the 11 countries that reported guidelines in the 2015 survey but did not respond in this survey, the countries in the sample represent 84% of all FCTC Parties known to have guidelines and 83% of Signatories. As we identified contacts in only three of the nine non‐Parties, and only one replied, we could not confirm guidelines status for most of the non‐Parties. Given that the majority of non‐Parties are low‐income countries, we believe it unlikely that many of these countries have guidelines yet.

The main limitation of this survey is that it was not practically possible to verify fully the accuracy of responses against the actual guidelines content. Wherever possible we asked respondents to send us a copy of, or a web link to, their guidelines with their completed survey to verify that the guidelines documents existed and were available to access on‐line or in another format. We have not inspected the content of the documents sent to us in detail, as that would go beyond the aims and scope of the study, and would also require fluency in a large number of languages, making it practically impossible.

Another limitation of the survey is that assessing guidelines implementation was beyond its scope.

As the majority of respondent countries were high‐income (twice as many as low‐ and middle‐income), this created imbalances in terms of sample sizes and limited the use of statistical regression analysis in making comparison across income categories.

### Guidelines content and key recommendations

As in our 2012 survey, most guidelines are evidence‐based and recommend brief advice, smoking cessation medications and intensive specialist support. Only half recommend HCWs setting an example by not using tobacco, consistent with the findings from the treatment survey 5. Given the high reported rates of tobacco use by HCWs and students in some countries 7, 8, 9, 10, this remains a seriously neglected area.

Most guideline recommendations did not differ significantly by countries’ income‐level, the exceptions being quitlines, intensive specialist support and some medications with data suggesting cost to be a factor. Given that these interventions require considerable resources this is perhaps not surprising, and is consistent with the finding from our survey of treatment provision, that UMIC and LMIC have less cessation support provision 5. It is also worth noting that our treatment survey found that, overall, only 23% of countries had national quitlines, very few in LMIC and none in LIC 5. The most important implication of this may be that as new mobile technology develops, text messaging systems and mobile phone applications may prove a far more cost‐effective way of offering support to whole populations. However, only 31% of guidelines are currently recommending text messaging for cessation.

Many guidelines still fall short of good practice in the writing process, as recommended in the FCTC Article 14 guidelines. Only around two‐thirds describe the writing and review process clearly, are peer‐reviewed and state clearly who funded the guidelines, and fewer than half include conflict‐of‐interest statements for all authors. The figures are a little better for endorsement by national professional associations (66%) and the government (67%). Lack of government backing and wide endorsement at national level could seriously undermine the authority of guidelines and undermine their implementation. Also, lack of transparency over authors’ conflicts of interest is likely to undermine the authority and credibility of guidelines, as is pharmaceutical industry funding.

Given understandable suspicion about the potential influence of pharmaceutical industry funding on guideline content, we are surprised to see that some guidelines are still pharma‐funded, and believe countries need to follow the FCTC Article 14 Guidelines more rigorously if they wish their guidelines to have credibility. The current New Zealand guidelines are a model of good practice in this respect 11. The FCTC Article 14 guidelines state that development of strategies to implement Article 14 should be protected from all actual and potential conflicts of interest.

### Guidelines updating and strategies for implementation

Nearly two‐thirds of guidelines were published or updated more than 5 years ago and 13% have not been updated since they were first published, some more than 10 years ago. A third recommend text messaging, the evidence base for which has grown since the last survey 12, 13, which illustrates the need for countries to update their guidelines periodically in order to reflect the evidence base, something clearly recommended in the FCTC Article 14 guidelines.

However, regular updating has cost implications and may be an issue for UMIC and LMIC, and it is interesting that referencing the Cochrane Library was less common in these countries. Although the residents of more than 100 low‐ and middle‐income countries currently have free access to the Cochrane Library 14 through a number of initiatives, this may not apply to all countries. However, this finding may reflect broader factors. For example, lower‐income countries are likely to have officials in charge who have to deal with all tobacco control, not just treatment, and possibly other topics as well, and simply have limited knowledge/expertise, limited time and possibly even poor internet access.

One way of dealing with this may be basing guidelines on those of other countries, which more than half say they do. However, national guidelines must be, by definition, for the country's own health‐care system, and must reflect the resources available. Tools are now available to help countries write or update their guidelines, including a library of national guidelines 15 and a review of the evidence base written especially for guideline development, which includes an affordability calculator for use within a country, using national data 13. This review was used by New Zealand to update their guidelines 11, and could serve as a simple and affordable approach other countries could adopt.

The FCTC Article 14 guidelines emphasize that national cessation guidelines should have a dissemination and implementation plan. In this survey, only 57% of countries say they have a strategy to disseminate their guidelines.

Dissemination strategies and action plans are crucial for the implementation of guideline recommendations. It is important that countries which have produced cessation guidelines without a dissemination strategy and implementation plan ensure that they are disseminated widely at national level, and that practical steps are taken to implement them in real life.

Overall our findings suggest that—as for treatment provision—producing tobacco cessation guidelines is a low priority. Twelve years after the FCTC came into force and 7 years after the Article 14 guidelines were adopted, most countries still do not have official national cessation guidelines. According to our global treatment survey, fewer than a third of countries have an official national tobacco cessation strategy 5. Although this survey was of cessation guideline content, we believe there is now a need for a survey of national cessation strategies to assess their coverage and content.

We suggest that countries need to look afresh at the recommendations of the FCTC Article 14 guidelines and implement them, using available tools to develop national strategies and guidelines quickly and affordably 2.

## Declaration of interests

M.R. is Director of the International Centre for Tobacco Cessation, which is funded by Pfizer and the InterAmerican Heart Foundation.

## Supporting information


**Table S1** Survey participants.

**Table S2** Survey respondent countries with guidelines.

**Table S3** Logistic regression model of guidelines recommendations and income level (Tables 1 and 3).

**Table S4** Professions, settings and client groups covered in the guidelines (Table 2).
