# Supplementary material for: A survey of tobacco dependence treatment guidelines content in 61 countries
Source: Addiction. 2018 Apr 16;113(8):1499–506. doi: 10.1111/add.14204 (PMC6099485; doi:10.1111/add.14204)
Supplement: Supplementary file 1 — Table S1 Survey participants. [file ADD-113-1499-s001.doc]

**Table E** 1 Survey participants

| **Countries** | **Contacts found (n)** | **Did not reply (n)** | **Did not have guidelines (n)** | **Completed the survey (n)** | **Response Rate, %** |
| --- | --- | --- | --- | --- | --- |
| Parties | 68 | 11 | 0 | 57 | 83.8 |
| Signatories | 6 | 1 | 1 | 4 | 83.3 |
| Non-Parties | 3 | 2 | 1 | 0 | 33.3 |
| **Total** | **77** | **14** | **2** | **61** | **79.2** |

[Parties = countries that signed and fully or partially ratified the FCTC](http://www.fctc.org/about-fca/tobacco-control-treaty/latest-ratifications/parties-ratifications-accessions#ratifications)

Signatories = c[ountries that have signed but not ratified](http://www.fctc.org/about-fca/tobacco-control-treaty/latest-ratifications/parties-ratifications-accessions#signed) the FCTC

Non-Parties =c[ountries that are neither signatories or Parties to the FCTC](http://www.fctc.org/about-fca/tobacco-control-treaty/latest-ratifications/parties-ratifications-accessions#neither)
