# Supplementary material for: A survey of tobacco dependence treatment guidelines content in 61 countries
Source: Addiction. 2018 Apr 16;113(8):1499–506. doi: 10.1111/add.14204 (PMC6099485; doi:10.1111/add.14204)
Supplement: Supplementary file 2 — Table S2 Survey respondent countries with guidelines. [file ADD-113-1499-s002.doc]

**Table E 2 Survey respondent countries with guidelines**

| **Country** | **FCTC Status** | **Date of FCTC signing/ratifying** |
| --- | --- | --- |
| Argentina | Signatory | 25-Sep-2003 |
| Armenia | Party | 29-Nov-2004 |
| Australia | Party | 27-Oct-2004 |
| Austria | Party | 15-Sep-2005 |
| Bahrain | Party | 20-Mar-2007 |
| Belgium | Party | 01-Nov-2005 |
| Bosnia and Herzegovina | Party | 10-Jul -2009 |
| Brazil | Party | 03-Nov-2005 |
| Brunei Darussalam | Party | 03-Jun-2004 |
| Canada | Party | 26-Nov-2004 |
| Chile | Party | 13-Jun-2005 |
| China | Party | 11-Oct-2005 |
| Czech Republic | Party | 01-Jun-2012 |
| Denmark | Party | 16-Dec-2004 |
| El Salvador | Party | 21-Jul-2014 |
| England | Party | 16-Dec-2004 |
| Estonia | Party | 27-Jul-2005 |
| Finland | Party | 25-Jan-2005 |
| France | Party | 19-Oct-2004 |
| Georgia | Party | 14-Feb-2006 |
| Germany | Party | 16-Dec-2004 |
| Greece | Party | 27-Jan-2006 |
| India | Party | 05-Feb-2004 |
| Iran | Party | 05-Nov-2005 |
| Italy | Party | 02-Jul-2008 |
| Japan | Party | 08-Jun-2004 |
| Jordan | Party | 19-Aug-2004 |
| Kyrgyzstan | Party | 25-May-2006 |
| Latvia | Party | 01-Feb-2005 |
| Malaysia | Party | 16-Sep-2005 |
| Malta | Party | 24-Sep-2003 |
| Mexico | Party | 28-May-2004 |
| Montenegro | Party | 23-Oct-2006 |
| Morocco | Signatory | 16-Apr-2004 |
| Netherlands | Party | 27-Jan-2005 |
| New Zealand | Party | 27-Jan-2004 |
| Northern Ireland | Party | 16-Dec-2004 |
| Norway | Party | 16-Jun-2003 |
| Pakistan | Party | 03-Nov-2004 |
| Panama | Party | 16-Aug-2004 |
| Paraguay | Party | 26-Sep-2006 |
| Poland | Party | 15-Sep-2006 |
| Portugal | Party | 08-Nov-2005 |
| Romania | Party | 27-Jan-2006 |
| Russia | Party | 03-Jun-2008 |
| Scotland | Party | 16-Dec-2004 |
| Singapore | Party | 14-May-2004 |
| Slovak Republic | Party | 04-May-2004 |
| Slovenia | Party | 15-Mar-2005 |
| Spain | Party | 11-Jan-2005 |
| Sweden | Party | 07-Jul-2005 |
| Switzerland | Signatory | 25-Jun-2004 |
| Thailand | Party | 08-Nov-2004 |
| Turkey | Party | 31-Dec-2004 |
| Ukraine | Party | 06-Jun-2006 |
| United Arab Emirates | Party | 07-Nov-2005 |
| United States of America | Signatory | 10-May-2004 |
| Uruguay | Party | 09-Sep-2004 |
| Venezuela | Party | 27-Jun-2006 |
| Vietnam | Party | 17-Dec-2004 |
| Wales | Party | 16-Dec-2004 |

[Parties = countries that signed and fully or partially ratified the FCTC](http://www.fctc.org/about-fca/tobacco-control-treaty/latest-ratifications/parties-ratifications-accessions#ratifications)

Signatories = c[ountries that have signed but not ratified](http://www.fctc.org/about-fca/tobacco-control-treaty/latest-ratifications/parties-ratifications-accessions#signed) the FCTC

Non-Parties =c[ountries that are neither signatories or Parties to the FCTC](http://www.fctc.org/about-fca/tobacco-control-treaty/latest-ratifications/parties-ratifications-accessions#neither)

*Source: United Nations Treaty Collection https://treaties.un.org/pages/ViewDetails.aspx?src=TREATY&mtdsg_no=IX-4&chapter=9&clang=_en*
