# Supplementary material for: A survey of tobacco dependence treatment guidelines content in 61 countries
Source: Addiction. 2018 Apr 16;113(8):1499–506. doi: 10.1111/add.14204 (PMC6099485; doi:10.1111/add.14204)
Supplement: Supplementary file 3 — Table S3 Logistic regression model of guidelines recommendations and income level (Tables 1 and 3). [file ADD-113-1499-s003.doc]

**Table E3 Logistic regression model of guidelines recommendations and income level (Tables 1 and 3)**

| **Guidelines recommendations** |  | **OR, (95%CI)** | **OR, (95%CI)** |
| --- | --- | --- | --- |
|  | HIC | UMIC | LMIC |
| Quitlines | 1 | 0.15 (95% CI 0.04 - 0.61) | 0.28 (95% CI 0.06 -1.23) |
| Intensive specialist support | 1 | 0.32 (95% CI 0.01 - 5.44) | 0.01 (95% CI 0.00 - 0.20) |
| Reference/refer to the Cochrane Library | 1 | 1.15 (95% CI 0.26 - 5.03) | 0.04 (95% CI 0.00 - 0.39) |
| Recommend bupropion | 1 | 0.42 (95% CI 0.06 - 2.84) | 0.10 (95% CI 0.02 - 0.61) |
| Recommend cytisine | 1 | 1.68 (95% CI 0.14 - 20.35) | 14.8 (95% CI 2.13 - 102.71) |
| Include conflict-of interest statements for all authors | 1 | 0.16 (95% CI 0.30 - 0.80) | 0.24 (95% CI 0.05 - 1.33) |

HIC= High income countries; UMIC= Upper middle income countries; LMIC=lower middle income countries
