# Supplementary material for: A survey of tobacco dependence treatment guidelines content in 61 countries
Source: Addiction. 2018 Apr 16;113(8):1499–506. doi: 10.1111/add.14204 (PMC6099485; doi:10.1111/add.14204)
Supplement: Supplementary file 4 — Table S4 Professions, settings and client groups covered in the guidelines (Table 2). [file ADD-113-1499-s004.doc]

**Table E4 Professions, settings, and client groups covered in the guidelines (Table 2)**

| **Guidelines recommendations** |  | **OR, (95%CI)** | **OR, (95%CI)** |
| --- | --- | --- | --- |
|  | HIC | UMIC | LMIC |
| Doctors? | 1 | 0.32 (95% CI 0.02 - 5.44) | 0.09 (95% CI 0.01 -1.16) |
| Nurses? | 1 | 0.33 (95% CI 0.07 - 1.49) | 0.07 (95% CI 0.01 -0.40) |
| Pharmacists? | 1 | 0.27 (95% CI 0.07 - 1.06) | - |
| Dentists? | 1 | 0.35 (95% CI 0.10 - 1.28) | 0.16 (95% CI 0.03 - 0.87) |
| Smoking cessation specialists? | 1 | 0.41 (95% CI 0.09 - 1.77) | 0.23 (95% CI 0.05 - 1.10) |
| Healthcare service managers? | 1 | 1.11 (95% CI 0.31 - 4.03) | 0.56 (95% CI 0.13 - 2.40) |
| Primary care? | 1 | 0.14 (95% CI 0.01 - 1.76) | 0.09 (95% CI 0.01 - 1.16) |
| Hospitals? | 1 | 0.88 (95% CI 0.22 -3.47) | 0.31 (95% CI 0.07 -1.39) |
| Mental health services? | 1 | 0.48 (95% CI 0.13 - 1.74) | 0.22 (95% CI 0.04 -1.20) |
| Addiction services? | 1 | 0.43 (95% CI 0.12 - 1.57) | 0.56 (95% CI 0.13 -2.40) |
| Prisons? | 1 | 0.32 (95% CI 0.04 - 2.87) | - |
| Smokeless tobacco users? | 1 | - | - |
| Pregnant tobacco users? | 1 | 0.43 (95% CI 0.12 - 1.54) | 0.25 (95% CI 0.05 -1.16) |
| Other? | 1 | 2.42 (95% CI 0.62 - 9.45) | 1.11 (95% CI 0.19 -6.39) |

HIC= High income countries; UMIC= Upper middle income countries; LMIC=lower middle income countries

Compared with HIC, LMIC were significanlty less likley to have smoking cessation guidelines for nurses, OR 0.07 (95% CI 0.01 – 0.40) and dentists OR 0.16 (95% CI 0.03 – 0.87). Logistic regression was not possible for pharmacists, prisons and smokeless tobacco users due to not enough values.
